# Establishing community structure and diversity within hydrothermal vent bacterial communities of the East Pacific Rise at 9°50′N

**DOI:** 10.1128/spectrum.00067-26

**Published:** 2026-03-09

**Authors:** Heather Fullerton, Nisarg Patel, Drew D. Syverson, Craig L. Moyer, Jason B. Sylvan

**Affiliations:** 1 Department of Biology, College of Charleston 2343, Charleston, South Carolina, USA; 2 Department of Earth, Environmental and Geographical Sciences, University of North Carolina at Charlotte 14727, Charlotte, North Carolina, USA; 3 Department of Biology, Western Washington University 1632, Bellingham, Washington, USA; 4 Department of Oceanography, Texas A&M University 14736, College Station, Texas, USA; Connecticut Agricultural Experiment Station, New Haven, Connecticut, USA

**Keywords:** hydrothermal vents, community structure, Zetaproteobacteria, Campylobacterota

## Abstract

Hydrothermal vents along the East Pacific Rise at 9°50′N support diverse microbial and animal communities driven by chemosynthesis. This study uses microbial growth chambers to investigate microbial colonization dynamics and community structure across multiple venting locations. Microbial growth chambers were deployed and incubated in vent fluids with distinct geochemical conditions for varying durations (2–257 days). Amplicon sequencing of SSU rRNA genes revealed that Campylobacteria, particularly *Sulfurimonas* and *Sulfurovum*, dominated across sites, in general, while Zetaproteobacteria were prevalent specifically in iron-rich environments. Community composition shifted over time, with an increased abundance of potential secondary producers and heterotrophs, including Alphaproteobacteria and Bacteroidia. Network analyses indicated dynamic microbial interactions, with community assembly influenced by temperature, sulfide, and iron availability. The findings suggest that microbial colonization at hydrothermal vents is a stochastic process shaped by environmental factors, with a subset of early colonizers persisting in developed communities. These results enhance our understanding of microbial succession in hydrothermal ecosystems and their role in biogeochemical cycling.

**IMPORTANCE:**

Established microbial communities are taxonomically and metabolically diverse, and this impacts their function within an ecosystem. The current study sheds light on patterns of community assembly at hydrothermal vents with varying chemistry in a small geographic range. Complex microbial communities formed rapidly and matured to include more potential heterotrophs at these locations. There were differences between sites, likely due to vent fluid chemistry. However, some taxa were found across all sites, even with these differences in chemistry. While common taxa were found across all sites, no one type was found to be consistently dominant, suggesting that microbial communities at hydrothermal vents may form randomly, depending on which bacteria arrive first and the local environment. However, chemistry still plays a big role in shaping these communities.

## INTRODUCTION

Seafloor hydrothermal vents are found along mid-ocean ridges, volcanic arcs, and back-arc spreading centers. These ephemeral habitats are plentiful in resources supporting diverse and rich assemblages of animal and microbial life. Hydrothermal vents create steep gradients due to the mixing of hot fluids composed of reduced chemicals with cold oxygenated seawater. This redox gradient allows for the proliferation of chemosynthetic microbes, which subsequently support animal diversity. In addition to supporting higher trophic levels, the resident chemosynthetic microbial biofilms could act as settlement cues for vent-endemic invertebrates (1, 2).

Hydrothermal vents are important to study for a variety of reasons. For example, they are excellent models for understanding disruption ecology—abrupt changes in chemistry and eruptive events can drastically impact microbial community structure and function and demolish existing microbial and animal communities (3–5). However, these events can also lead to the creation of new habitats and changes in adjacent animal assemblages (5–7). Vent fluids are enriched in reduced compounds that impact surface ocean primary productivity and global biogeochemical processes (8, 9). Beyond their ecological and geochemical roles, hydrothermal vents also provide a modern analog for early Earth environments, offering critical clues to how life may have originated and evolved under extreme conditions (10).

At all hydrothermal vents, chemosynthetic bacteria are foundational community members and can be found free-living and in symbiosis with vent invertebrates. The chemosynthetic microorganisms at vents primarily derive energy from hydrogen, sulfide, or iron oxidation, which can be coupled with autotrophic carbon fixation (11, 12). Hydrogen oxidation has been identified across diverse classes of hydrothermal vent bacteria (13, 14), including *Caminibacter* species, a member of the phylum Campylobacterota that has been isolated from hydrothermal vents and grown chemosynthetically through hydrogen oxidation (15, 16). Other members of the phylum Campylobacterota, such as *Sulfurimonas* and *Sulfurovum,* have been identified as key players in sulfur oxidation at numerous vent locations (17, 18). Within the phylum Pseudomonadota (previously known as the phylum Proteobacteria) (19), organisms within the class Zetaproteobacteria have been identified as iron-oxidizers and dominant community members at iron-rich vents (20, 21). One isolate of Zetaproteobacteria, *Ghiorsea bivora*, can oxidize iron and hydrogen to fuel chemosynthetic growth (13). Other Pseudomonadata have been identified as important chemoautotrophic members of hydrothermal vent microbial mat communities, including the genera *Beggiatoa* and *Thiomicrospira* (22, 23). While the chemosynthetic Campylobacteria and Zetaproteobacteria are globally distributed, microbial communities at hydrothermal vents are shaped locally (24–26). In the case of Zetaproteobacteria, network analysis revealed a competitive relationship amongst oligotypes of the most abundant Zetaproteobacterial OTUs across two disparate hydrothermal vent regions of the Pacific Ocean, which may indicate that speciation is occurring (25).

Ecological interactions are defined by their effects on each party: beneficial, harmful, or neutral (27). In microbial community analysis, keystone taxa are central to the function of an ecosystem and are identified by co-occurrence patterns or microbial network-based analyses (28). Network analysis can also identify commensal, competitive, or cooperative relationships in a microbial community (25, 29). These types of analyses for microbial communities primarily rely upon amplicon sequencing. Oligotyping and amplicon sequence variant (ASV) methods were developed to increase biogeographic resolution by distinguishing even single-nucleotide differences, providing finer insights into the potential ecological differentiation of ASVs in natural environments (30, 31). A targeted SSU-based amplicon survey from Kama’ehuakanakloa Seamount showed that using the V4V5 primer set was the most efficacious when examining iron-dominated microbial mats that are higher in Zetaproteobacteria in terms of detection, but suggested that the V3V4 primers might be better suited for discriminating among sulfur-dominated habitats that are higher in Campylobacterota (32). The ASV approach, regardless of primers used, includes error correction and is a more sensitive metric than OTUs as indicators for biogeography (33–35). Network approaches using ASV sequencing have been increasingly applied to microbial communities to understand complex interactions and to elucidate microbial function (36, 37). One such study used microbial growth chambers (MGCs) to examine colonization dynamics and community structure at hydrothermal vents at sites from Kama’ehauakanloa Seamount (Hawaiian Hotspot), Axial Volcano (Juan de Fuca Ridge), and Magic Mountain (Explorer Ridge), with network analysis providing critical insights into microbial interactions, particularly among Campylobacteria and Zetaproteobacteria, and revealing the influence of environmental factors such as temperature and dissolved iron on community assembly and biogeographic distribution (38).

Both abiotic and biotic factors influence the formation of complex microbial communities, which are crucial for the overall health and stability of ecosystems. To elucidate how microbial communities form at hydrothermal vents across multiple venting temperatures within a small geographic range, MGCs were incubated in or near fluid flow at the seafloor of 9°50′N East Pacific Rise (EPR), a fast-spreading mid-ocean ridge hydrothermal system that has been well-studied since its discovery; but for which, significant discoveries of spatial and temporal differences in magmatic, volcanic, and hydrothermal activity and the surrounding biosphere are still being made (39, 40). These MGCs contain silica wool inside a section of plexiglass tubing as a fresh surface to facilitate microbial attachment and growth. The silica wool is enclosed in nylon mesh across the opening of the tube to allow fluid flow while preventing grazing. The use of MGCs enables the exploration of targeted questions regarding the dynamics of colonization and its impact on the resulting diversity of microbial communities. Additionally, using an inert substrate for microbial attachment removes the substrate attachment geochemistry as a variable in colonization. This is similar to other colonization experiments, where a chamber provides surfaces for attachment (41, 42) and where the transition from seafloor to a laboratory environment has minimal impact (43).

EPR 9°50′N is characterized by multiple venting locations, where the vent fluid temperature and chemistry have been recorded to vary significantly on short timescales (44). This hydrothermal system has experienced three major observed eruption events on decadal timescales: 1991, 2005/2006, and 2025 (44–50). After each eruption, the temperature of the vent fluid is observed to decrease, while the dissolved sulfide-to-iron concentration ratio increases. This is followed by a long-term period during which vent fluids are shown to slowly increase in temperature, while the dissolved sulfide/iron concentration ratios decrease, until just prior to the next eruptive event. In this dynamic habitat, both active and inactive venting sites play a crucial role in supporting microbial biogeochemical cycles and providing habitats for animals (5, 51). To test the impact of vent temperature while examining community changes over time, the diversity of active hydrothermal vent environments was leveraged at EPR 9°50′N to test bacterial colonization dynamics by deploying MGCs in high-temperature (>150°C) and low-temperature (<40°C) venting fluids for short (<10 days) and long (>100 days) durations. This approach enabled testing of how temperature, geochemistry, and time influence the development of nascent bacterial populations in vent environments. While Campylobacteria were detected across all sites, the influence of chemistry and time was found to influence the presence of Zetaproteobacteria, Gammaproteobacteria, Alphaproteobacteria, and Bacteroides. This study provides important insights into the driving factors of bacterial colonization in dynamic and ephemeral environments.

## MATERIALS AND METHODS

### Sample collections and processing

MGCs were constructed as previously described (38) and deployed at eight venting locations (M Vent, Riftia Field, Teddy Bear, Tica, P Vent, Riftia Mound, Bio9, and Marker 28; Fig. S1) along the EPR 9°50′N that varied in temperature from 4°C to 376°C using the human-operated vehicle (HOV) Alvin during R/V Atlantis cruise AT42-06 in December 2018, March 2019 (AT42-09), and December 2019 (AT42-21). Prior to deployment, the MGCs were free of biomass, and the MGCs were placed on top of fluid emanating from sulfide structures or from the seafloor. The Alvin temperature probe was used to record temperature at the MGC placement location in all cases, both prior to deployment and upon recovery at the end of the incubation. Upon recovery of the MGCs at the seafloor, they were placed into a sealed box and subsequently transported to the surface for recovery. Once MGCs were recovered on board, the silica glass wool of each MGC was removed and then frozen at −80°C until further processing. Cell biomass was later separated from the silica glass wool, and DNA was extracted as previously described (38).

Established microbial mats were collected during the March 2019 cruise using an impeller pump-driven suction sampling device on HOV Alvin. After collection, samples were mixed with an equal volume of RNALater and stored for 24 h at 4°C before being frozen at −80°C until further processing. DNA was extracted as previously described (52). All DNA was quantified by a Qubit 4.0 fluorometer using high-sensitivity reagents (ThermoFisher Scientific, Waltham, MA).

### DNA sequencing and bioinformatics analysis

The bacterial SSU rRNA gene was amplified by PCR using V3–V4 primers (52, 53). The resulting amplicons were sequenced using a MiSeq (Illumina, San Diego, CA) following the manufacturer’s protocol to generate 2 × 300 bp reads. After sequencing, reads were trimmed of primers using CutAdapt (54).

Amplicon sequence data processing and analysis were performed using a pipeline involving the DADA2 (33) and Phyloseq (55) packages in R (version 4.4.0). ASVs were created using default commands in DADA2, followed by removing chimeric sequences using the removeBimeraDenovo function and the consensus method. The resulting ASVs table dreived from 57 samples and contained 34,357 ASVs. The SILVA SSU rRNA database (v 138.1) was used for the taxonomic classification of ASVs. Taxonomic composition, diversity analyses, and statistical comparisons were conducted using the vegan, Phyloseq, DESeq2, and Microbiome packages (55–58). Alpha diversity differences by duration of incubation were tested by analysis of variance (ANOVA) if the data were normally distributed and by Kruskal-Wallis for non-normal data. Differences in beta diversity were tested using permutational analysis of variance (PERMANOVA), which was preceded by PERMDISP2 to test for multivariate homogeneity of group dispersions. Visualizations were completed with ggplot2 (59). Non-metric multidimensional scaling (NMDS) ordinations based on Bray-Curtis distances were performed on the relative abundance data using the ggordiplot function (60). Ordination results included 95% confidence intervals for each sample group, with paths delineating group boundaries. For higher resolution, Zetaproteobacteria OTUs were determined using ZetaHunter (61, 62).

A network was constructed using the netConstruct function in the NetCoMi package in R (63), with Pearson correlation selected as the association measure, utilizing a phyloseq object that was filtered to retain all ASVs with a relative abundance of at least 1.0% relative abundance. Count data were centered log-ratio transformed, and zero values were handled using the “pseudo” method with a pseudocount of 0.5. Associations were retained if the absolute Pearson correlation coefficient exceeded 0.4, producing a weighted adjacency matrix. The constructed network was then analyzed using netAnalyze, with the “cluster_fast_greedy” method applied for modular clustering (63). The relative abundance of ASV determined the node sizes, and singletons were removed. Cluster hub ASV sequences and their closest matches, as identified by BLAST with the refseq RNA database, were aligned using Muscle (64) and trimmed to remove poorly aligned or gap-rich regions with decipher’s MaskAlignment (65). The trimmed alignments were converted to DNAbin format, and a phyDat object was generated for phylogenetic analysis using phangorn (66). Pairwise distances were calculated using maximum likelihood, and a neighbor-joining tree was constructed. The final tree was midpoint-rooted and visualized with ggtree (67).

### Chemistry

The composition of fluids sampled from the EPR 9°50′N hydrothermal system is derived from previously published data (Fig. S2) (68). For the purpose of vent chemistry, the composition of low-temperature fluids (<300°C) considered in this study is representative of hydrothermal fluids with Mg^2+^ concentration values less than seawater but [Mg^2+^] >0 and of endmember hydrothermal fluids representing pristine hydrothermal fluids venting from the seafloor. The composition of high-temperature fluids (>300°C) considered is only representative of pristine endmember hydrothermal fluids without any effects from seawater mixing (i.e., [Mg^2+^] = 0).

## RESULTS

**Fig 1:**
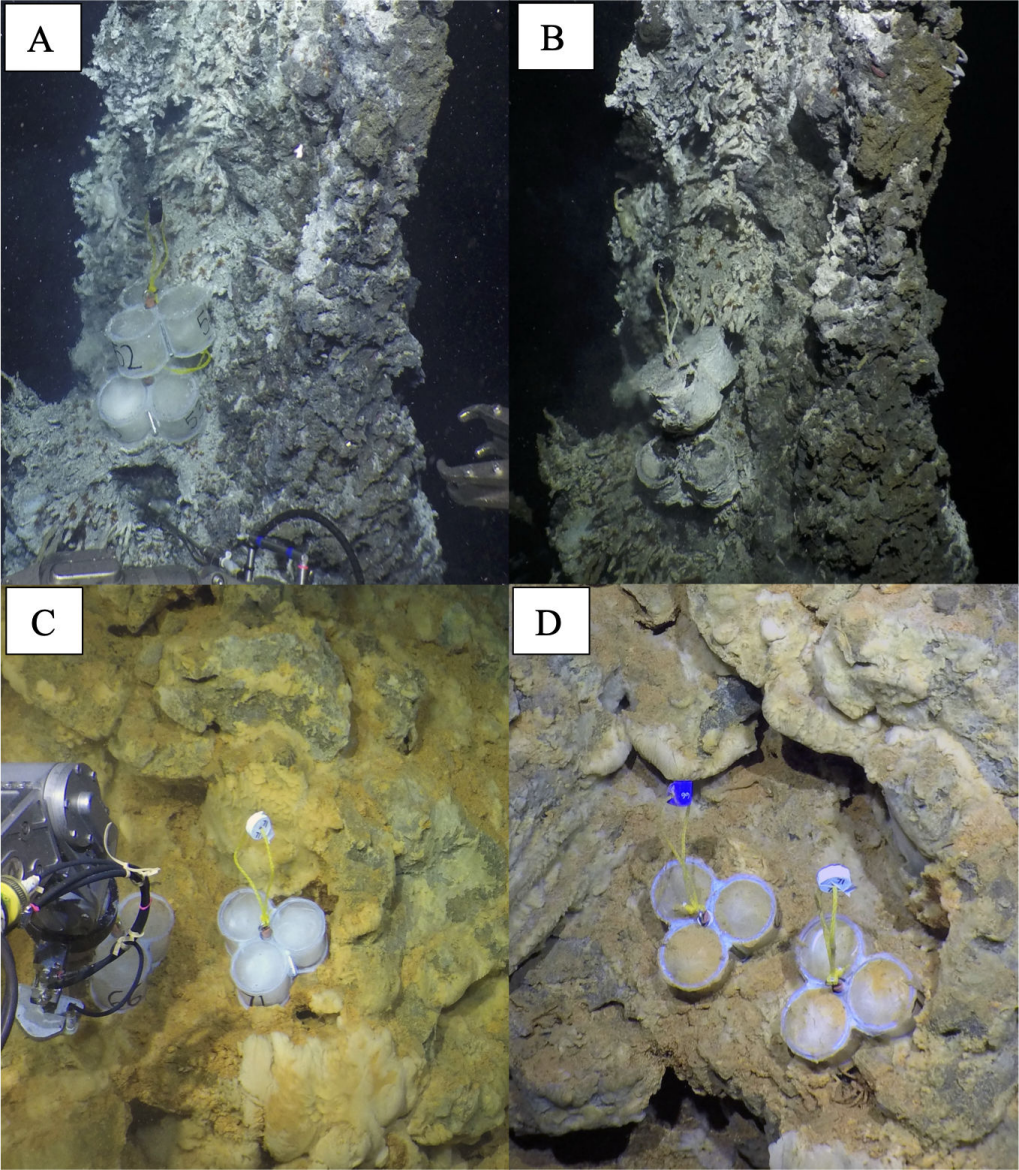
Example images of MGCs from two EPR 9°50′N vent locations. (**A**) MGCs at the time of placement on a spire at Tica and (**B**) after 5 days of *in situ* incubation. (**C**) MGCs at the time of placement at Riftia Field and (**D**) after 4 days of *in situ* incubation.

MGCs were placed and recovered during three research expeditions to the EPR 9°50′N in December 2018, March 2019, and December 2019. These MGCs were incubated for two to 257 days. Short-duration incubations, referring to the deployment of MGCs with incubation periods of less than 10 days, were conducted at all vent sites sampled here. At all sites, excluding Tica and Riftia Field, incubations were additionally conducted for durations greater than 100 days, referred to as long-duration incubations (Table 1; Table S1). The three longest incubated MGCs were at Marker 28 and P Vent, and the four shortest incubated MGCs were at Tica and Teddy Bear. Of the vent sites, Bio9, P Vent, M Vent, and Tica have focused flow with high venting temperatures (>150°C). On the other hand, Marker 28, Riftia Mound, Riftia Field, and Teddy Bear have more diffuse flow with lower venting temperatures (<40°C). All MGCs exhibited a color change over the incubation period, with color depending on where the MGCs were incubated. For example, at Tica, where vent fluids have high dissolved sulfide concentrations, the MGCs were covered in a white biofilm, and the silica wool’s opacity increased (Fig. 1). In contrast, at Riftia Field, where vent fluids have relatively low dissolved sulfide concentrations, the MGCs increased in opacity to orange rather than to a white biofilm (Fig. 1).

**TABLE 1:** Summary of collected MGCs with temperature and incubation length characterization^*a*^

Location name	Location	Mean vent temperature with standard deviation (°C)	Vent temperature	MGCs incubated <10 days	MGCs incubated >100 days
Bio9	9°50.30 N 104°17.48 E	334.8 ± 44.91	High	7	2
P Vent	9°50.28 N 104°17.47 E	324.38 ± 66.98	High	8	3
M Vent	9°50.80 N 104°17.59 E	184.43 ± 136.15	High	3	2
Tica	9°50.40 N 104°17.49 E	117.33 ± 20.17	High	6	0
Marker 28	9°50.16 N 104°17.45 E	9.50 ± 4.08	Low	5	4
Riftia Mound	9°50.29 N 104°17.48 E	29.00 ± 13.17	Low	4	4
Teddy Bear	9°50.5 N 104°17.51 E	10.65 ± 3.16	Low	2	2
Riftia Field	9°50.72 N 104°17.58 E	15.85 ± 0.21	Low	2	0

^*a*^ Temperature measurements were collected *in situ* during sample collection at each hydrothermal vent.

**Fig 2:**
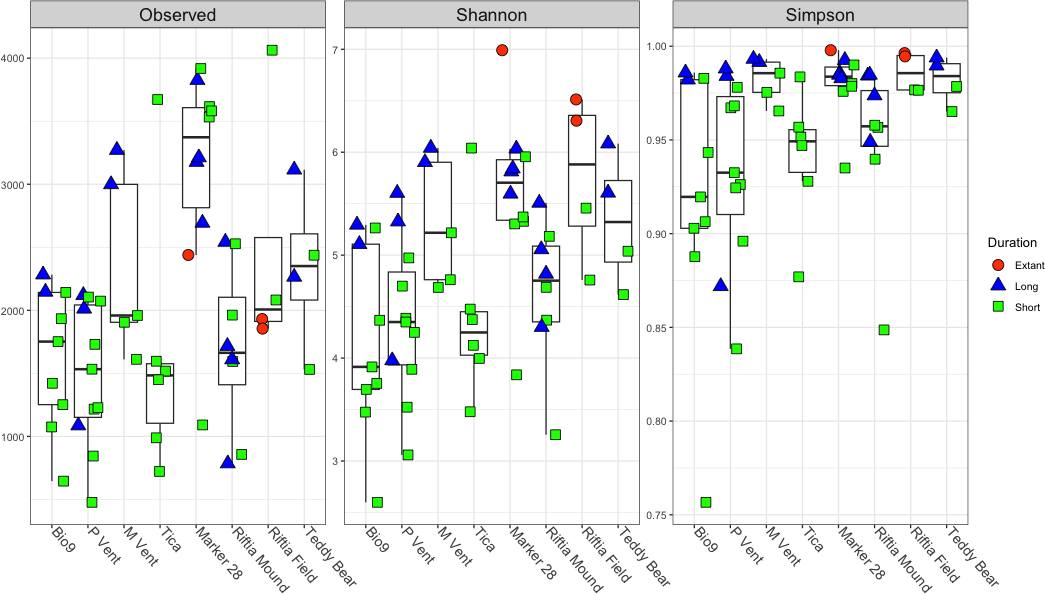
Boxplot of observed ASVs, Shannon diversity index, and Simpson index. Bio9, P Vent, M Vent, and Tica are high-temperature vent sites, and Marker 28, Riftia Mound, Riftia Field, and Teddy Bear are low-temperature vent sites.

Relatively high diversity was reached very quickly in the short-term deployed growth chambers (Fig. 2). The MGCs incubated at lower temperature vents (<40°C) had higher diversity than the ones incubated at high temperature vents, as determined by a two-sample *t*-test between the MGCs incubated at high and low temperature vents for the observed ASVs and the Shannon and Simpson indices (Table S2). There was no statistical difference in alpha diversity between the MGCs incubated for less than 10 days and those incubated for more than 100 days, regardless of temperature, except for the MGCs from M Vent (Table S3), where ANOVA showed significant differences in observed ASVs and Shannon diversity as a factor of the incubation but not for Simpson’s diversity. In addition to the MGCs, established microbial mats were collected at Marker 28, and there was no significant difference by incubation period by ANOVA for Shannon diversity or Kruskal-Wallis for observed ASVs and Simpson’s diversity. Riftia Field and Tica could not be tested due to low replication of MGCs and incubation durations at these sites.

**Fig 3:**
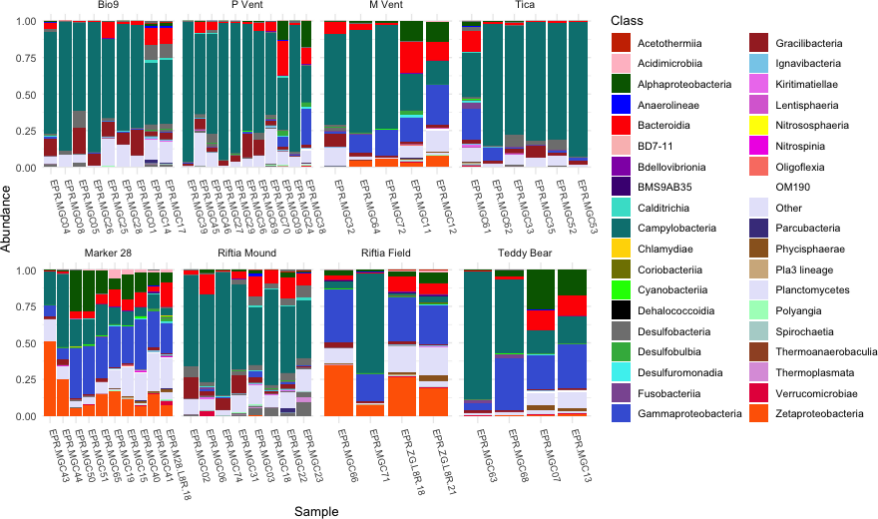
Stacked bar graph showing the taxonomic distribution of bacterial classes with relative abundance greater than 0.1%; all classes at or below 0.1% are combined into the “Other” category. Samples are ordered on the *x*-axis from shortest to longest incubation period.

The distribution of microbial classes varied across sampling locations (Fig. 3). Community composition also shifted over time to have a greater relative abundance of potential secondary producers and heterotrophs, including members of Bacteroidia, Alphaproteobacteria, and Gammaproteobacteria. The majority of the sites and MGCs were dominated by bacteria of the class Campylobacteria (Fig. 3). In five MGCs from Riftia Mound, two from Teddy Bear, and one each from Marker 28 and Riftia Field, the Campylobacteria abundance was greater than 50%. The genera *Sulfurimonas* and *Sulfurovum* were identified in all the MGCs except MGC41, which was incubated at the high iron site, Marker 28 (Fig. S3). *Caminibacter,* another genus within the class Campylobacteria, was more abundant within the MGCs incubated in Tica than in any of the other locations and composed 21.67% of the total community in MGC52. Both MGC61 and MGC62 fell off the spire where they had been placed at some point during their 2-day incubation, resulting in MGC61 having a different community composition than the other MGCs at Tica. Notably, these two MGCs had a much lower relative abundance of *Caminibacter*.

The MGCs incubated at Marker 28 and Riftia Field showed a relatively high abundance of Zetaproteobacteria. Within this class, Zeta OTU1, Zeta OTU2, and Zeta OTU14 were the most prevalent, composing 6.57%, 11.87%, and 10.70% of the total community, respectively (Fig. S3). Zeta OTU9 was a minor community member at the low temperature sites; however, it composed 2.7% of the total MGC community for MGC12, incubated at M Vent.

The MGCs at sites with lower temperatures and more diffuse fluid flow had a higher abundance of Gammaproteobacteria than the MGCs at high-temperature vent locations. Three MGCs, MGC44, MGC66, and MGC68, had the highest relative abundance of Gammaproteobacteria, and these were short-term incubations at Marker 28, Riftia Field, and Teddy Bear, respectively. The most abundant Gammaproteobacterial taxa represented were the cosmopolitan heterotrophs *Pseudomonas* spp. and various genera within the order Alteromonadales. Other genera within the Gammaproteobacterial class, such as *Marinobacter*, *Halomonas, Methyloprofundus,* and *Alcanovorax,* were identified within the MGCs. Alphaproteobacteria were also more abundant, on average, at the lower temperature sites than at the higher temperature ones. ASVs classified as Rhodobacteraceae, Sphingomonadaceae, and Hyphomonadaceae were most abundant in these MGCs, most often only classifiable to the family level.

Relative abundances of Alphaproteobacteria and Bacteroidia were significantly higher in the long-duration incubations at both low and high temperature locations (Fig. S4). Bacteroidia had a higher abundance in the MGCs that were incubated for more than 100 days (Fig. S5). The MGCs incubated at Bio9, Tica, and Riftia Mound had a relatively low abundance of Bacteroidia, and at Tica, the relative abundance of Bacteroidia decreased over time (Fig. S5). This is in contrast to the other MGCs, where the relative abundance of Bacteroidia increased with incubation time. Additionally, the incubation time period was a strong predictor for increasing Alphaproteobacteria at P Vent and M Vent, where a few ASVs classified as Rhodobacteraceae increased in relative abundance in longer incubations, and at Teddy Bear, where unidentified Rhodobacteraceae and *Robiginitomaculum* increased in relative abundance with time. The incubation time period was also a strong predictor for increasing Bacteroidia at M Vent and Teddy Bear (Fig. S5). This indicates that Campylobacteria settle early, whereas other taxa, such as Alphaproteobacteria and Bacteroidia, settle later.

The MGCs showed overlap and diversity of ASVs between incubation temperatures and durations. The high temperature, short-duration, and long-duration incubation shared 3,866 ASVs (Fig. S6A). More ASVs, 5,479, were shared between the low temperature short-duration and long-duration incubations (Fig. S6B). At both the high and low vent temperature locations, there were more unique ASVs within the short-duration incubations than in the long-duration incubations. Across the two temperatures and the two incubation durations, 2,707 ASVs were shared (Fig. S6C). Class identification of the shared ASVs is listed in Table S4.

**Fig 4:**
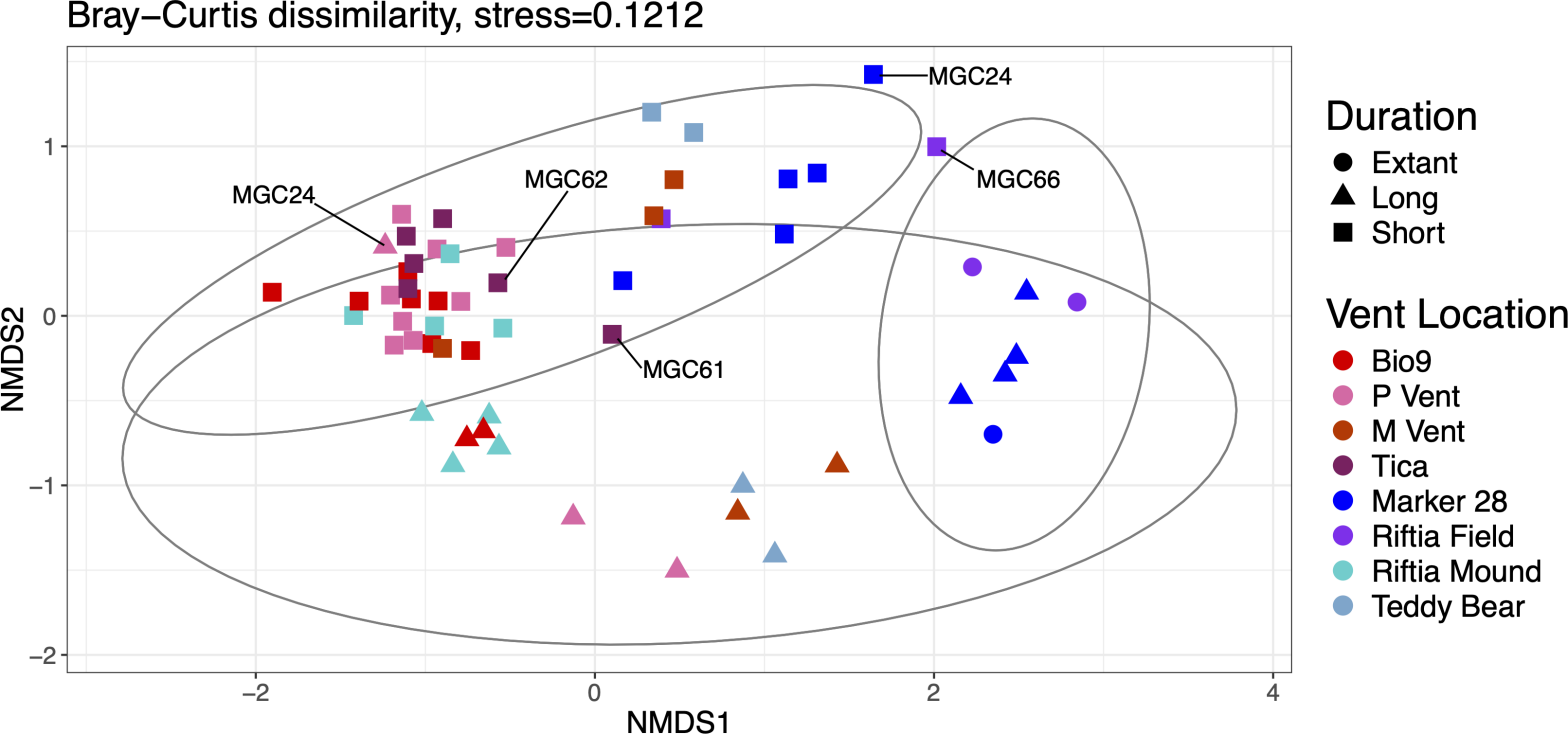
Non-metric multidimensional scaling plot generated using Bray-Curtis dissimilarities for the MGC microbial communities. Ellipses represent 95% confidence intervals for the incubation duration.

The relationship among the MGCs' community composition was examined through the Bray-Curtis dissimilarity index. This community analysis separated the MGCs by incubation duration (Fig. 4). The three extant microbial mats clustered with MGCs incubated for a long duration and at the lower temperature sites. Three MGCs (MGC24, MGC43, and MGC66) were not contained within their described duration confidence interval (Fig. 4). MGC24 was incubated at P Vent for 119 days. This MGC has a higher abundance of Campylobacteria than the other long-duration MGCs at P Vent, which could explain why it clustered near the short-duration and high-temperature MGCs. MGCs 43 and 66 had short-duration incubations and were located at Marker 28 and Riftia Field, respectively. Both of these MGCs had the highest relative abundance of Zetaproteobacteria. There was less of a separation when examining temperature (Fig. S7), due to the MGCs incubated at Riftia Mound, which clustered closer to those incubated at high-temperature locations. These MGCs contained a high proportion of Campylobacteria and a low abundance of Gammaproteobacteria, unlike the other low-temperature MGCs. PERMANOVA analysis revealed the location and the continuous variables of duration (days) and temperature (°C) to be significant factors (Table S5). Using categorical variables for temperature and duration failed the beta-dispersion test; therefore, the PERMANOVA could not be completed. Additionally, this suggests these groups have significantly different dispersions or unequal group spread. In contrast, using the continuous variable doesn’t create artificial boundaries, which would cause the beta dispersion test to fail. MGC61 and MGC62 became dislodged from the chimney structure during incubation; both were still within their incubation period confidence interval, but MGC61 was nearer to the low temperature MGCs.

**Fig 5:**
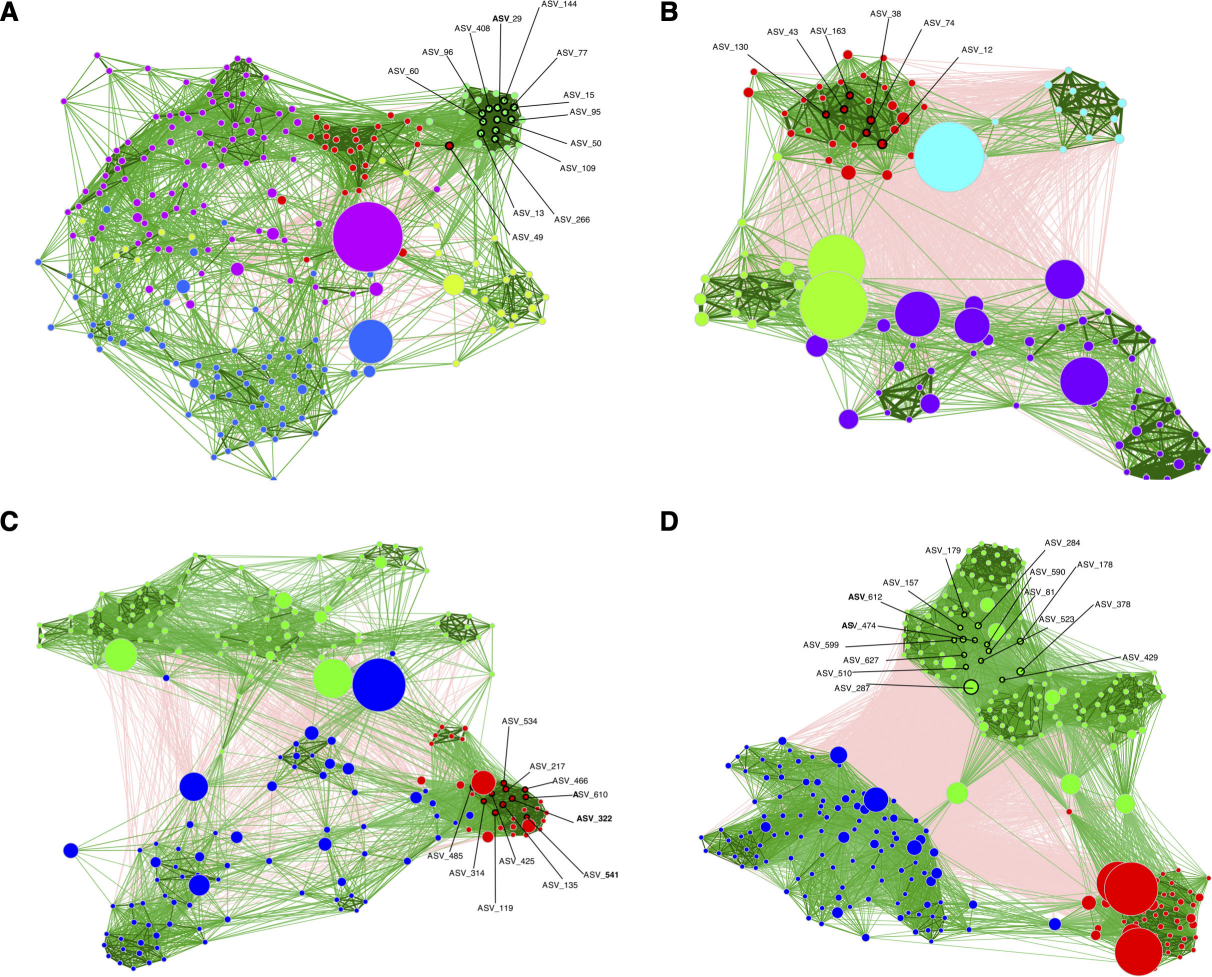
Microbial association networks for (**A**) MGCs incubated for a short duration at high temperature vents, (**B**) MGCs incubated for a long duration at high-temperature vents, (**C**) MGCs incubated for a short duration at low-temperature vents, and (**D**) MGCs incubated for a long duration at low-temperature vents. Green edges indicate a positive association, whereas red edges indicate a negative one. Line thickness indicates the strength of the association. Each point represents a node or a unique ASV, where size reflects relative abundance. Nodes with ASV labels and a black outline are network hubs. Colors of the nodes represent network clusters.

To assess changes in community interactions, the MGCs were subset into four groups based on incubation duration and vent temperature for network analysis. This analysis revealed different interactions across vent temperature and incubation time for the MGCs (Fig. 5). In network analysis, connectivity describes how strongly nodes are linked, while modularity reflects how clearly the network is organized into distinct clusters or communities. Networks created from the long-duration MGCs tended to have higher modularity and connectivity (Table S6). In contrast, the short-duration MGCs had lower node connectivity and higher modularity, indicating fewer cross-group links and potentially lower network resilience. The short-duration MGCs also displayed lower edge density, suggesting weaker correlations among ASVs. The proportion of positive edges represents co-occurring relationships; among the four networks, the short-duration, high-temperature network had the highest percentage of positive edges (95.24%), whereas the long-duration, high-temperature network had the lowest (58.13%).

There were five distinct network clusters for the short-duration, high-temperature MGCs and four for long-duration, high-temperature MGCs. Both duration MGCs at the low-temperature vents had three clusters. In addition to having the most clusters, the short-duration, high-temperature had the most ASVs (243), most of which were classified as Campylobacteria (146 ASVs). For MGCs deployed at the same sites with longer incubations, ASVs classified as Campylobacteria were still the most abundant, but no longer composed over half of the ASVs within the network. This pattern was the same for the low-temperature incubations. Campylobacteria are the most represented ASV in the short-duration incubations, and their relative abundance decreases in the long-duration incubations (Fig. S8). Regardless of incubation duration, Gammaproteobacteria and Zetaproteobacteria are more highly represented in the low temperature networks than in the high temperature networks.

Cluster hubs may represent ASVs that are ecologically important, and no hubs were shared among MGCs for different vent temperatures or incubation durations. As the microbial community matured, the community became more resilient and less connected to the putative primary producers, the Campylobacteria. Ten Campylobacteria and three Gammaproteobacteria ASVs were identified as cluster hubs in the high temperature, short-duration incubations (Table S7). These ASVs were closely related to *Sulfurimonas autotrophica* and *Sulfurovum riftiae* (Fig. S9). In contrast, for the high temperature, long duration incubations, one ASV of Caldisericia, Bacteroidia, Thermotogae, and Desulfobacteria, and two ASVs unidentified to the class level were the hubs. Campylobacteria (10 ASVs) and one Fusobacteria were identified as hubs for the low temperature, short-duration incubation Table S7; Fig. S8). The hubs for the low temperature long-duration MGCs incubated at the low temperature vents were Alphaproteobacteria (two ASVs), Campylobacteria (one ASV), Gammaproteobacteria (six ASVs), and one ASV unidentified to the class level (Table S7; Fig. S8).

## DISCUSSION

Hydrothermal vents are dynamic and ephemeral habitats where evidence from metagenomic and amplicon analyses of microbial mats and plumes indicates that bacteria predominate over archaea in hydrothermal systems (32, 69, 70). Previous research at the EPR 9°50′N hydrothermal system has shown the development of microbial biofilms on sterile basalts to occur within 4 days, and analysis after a 9-month incubation showed most OTUs settle early and persist (71). However, a study of metal sulfides at Tica Vent showed that alpha diversity varied depending on the mineral surface chemistry in short-term (13-day) incubations (72). Therefore, this study aimed to elucidate bacterial colonization dynamics at hydrothermal vents without the influence of surface chemistry as a driver at high or low temperature fluids within a small geographic range, the EPR 9°50′N vent area. The use of silica wool in this study isolates the effects of vent fluid chemistry and temperature from those of substrate reactivity, allowing us to assess the extent to which community assembly is driven by fluid composition rather than mineral interaction. The similarity among the established mats and the MGC communities supports this observation. Previous research at Kama‘ehuakanaloa Seamount used MGCs to assess colonization dynamics and found that Zetaproteobacteria were the dominant member within these chambers when the mean temperature was ≤40°C. At the same time, Campylobacteria dominated when the temperature was ≥71°C (73). More recently, an analysis of MGCs that were incubated at multiple venting locations at Axial Seamount, Magic Mountain, and Kama‘ehuakanaloa Seamount revealed the influence of chemistry on colonization dynamics (38). High sulfide concentrations characterize vent fluids at Axial Seamount and Magic Mountain, whereas Kama‘ehuakanaloa Seamount contains high dissolved iron and lower sulfide concentrations (74–76). Amplicon analysis from Axial Seamount and Magic Mountain MGCs showed a dominance of Campylobacteria, whereas Zetaproteobacteria primarily colonized Kama‘ehuakanaloa Seamount MGCs (38). Additionally, this study showed that the location strongly influenced the microbial community even after an eruptive event when temperature and sulfide concentration increased. Using different types of colonization chambers, Campylobacteria were found to be the dominant members, providing insights into which organisms can colonize newly formed surfaces (41, 42).

The results presented here expand upon previous studies and further support that location and chemistry influence microbial community composition (38, 77, 78). In this study, the geochemistry varied with respect to reduced iron and sulfide concentrations in a relatively small geographic range, yet there was a set of ASVs observed across all sites. The shared ASVs among durations suggest some ASVs settle early and persist, and there is a local influence separate from the vent fluid temperature. However, there were more unique ASVs than shared, perhaps indicating high turnover and time preference for some ASVs. All sites had known sulfur-cycling genera of Campylobacteria. The most abundant genera of Campylobacteria across all MGCs were *Sulfurimonas*, followed by *Sulfurovum* and *Caminibacter*. Genomic analysis of the globally distributed *Sulfurimonas* shows evidence of allopatric speciation, and the primary driver of the genetic variation is likely geographic distance (24). Comparisons of hydrothermal niches suggest that *Sulfurovum* grows mainly in diffuse flows (79), whereas the distribution of *Sulfurimonas* is considered more cosmopolitan (80). This is supported here by the high abundance of *Sulfurimonas* across the MGCs. *Sulfurimonas autotrophica*, the type species for the genus, was isolated from the Mid-Okinawa Trough hydrothermal field and grows chemosynthetically with sulfide, elemental sulfur, and thiosulfate as electron donors (81). Meanwhile, *Caminibacter* spp. are known to grow chemosynthetically by hydrogen oxidation and reduce sulfur, and the type species, *Caminibacter hydrogeniphilus*, was originally isolated from the EPR (15, 16). Hydrogen concentrations are not as regularly recorded as sulfide (68) at Tica and P Vent, which had some of the highest relative abundance of *Caminibacter*. However, the high abundance of *Sulfurovum*, *Sulfurimonas*, and *Caminibacter* supports that both sulfur-cycling and hydrogen-oxidation are important metabolisms for the bacterial communities in these hydrothermal fluids.

Zetaproteobacteria have been identified in marine habitats that range in reduced iron (Fe^2+^) concentrations. However, not all OTUs show equal distribution, likely due to variations in their physiology. All Zetaproteobacteria have evidence for microaerobic autotrophic growth on Fe^2+^ through growth and genomic studies (21, 82–84). Zeta OTU2 was the most abundant across the MGCs, and since its identification, this Zeta OTU has been identified globally as associated with hydrothermal microbial mats and within metal corrosion incubations (61, 85). Zeta OTU1 through Zeta OTU4 are considered cosmopolitan. A cultured representative from only Zeta OTU3 exists, and it was found to oxidize Fe and produce stalks similarly to *Mariprofundus ferooxidans* (25, 86). The physiology of the other cosmopolitan Zeta OTUs has been inferred from related isolates (21, 84). The second most abundant Zetaproteobacteria was Zeta OTU14. This OTU has been found to be associated with bioturbated sediments of coastal areas and was isolated under microaerobic conditions (87, 88).

Zeta OTU9 was detected at M vent and Riftia Field. Since MGC12 incubated at M vent had Zeta OTU9, this perhaps indicates changing temperature and geochemical conditions during the long duration of *in situ* incubation. This is also reflective of the lower temperature recorded in both 2014 and 2018 (78). However, subsequent MCG placements and other expeditions at M Vent recorded temperatures over 300°C (46). In addition to growing on dissolved Fe^2+^, *Ghiorsea bivora*, a cultured isolate classified as Zeta OTU9, has been shown to grow chemosynthetically by oxidizing H_2_ and has been identified in microbial mats on chimneys with temperatures around 50°C (13, 89). It is unknown if this Zeta OTU is growing by H_2_ or Fe^2+^ oxidation at the EPR 9°50′N hydrothermal system, and Zeta OTU9 has a different distribution than the H_2_-oxidizing *Caminibacter*. However, this is likely due to differences in optimal growth temperatures rather than substrate limitations, since isolated Zetaproteobacteria are mesophiles (62). This study expands the known biogeography of Zeta OTU09, which has now been detected at multiple hydrothermal sites and in subseafloor environments (62, 90, 91), further supporting the notion that it should be considered cosmopolitan.

The current study showed that microbial diversity is quickly established, and as the community matures, potential heterotrophs, such as Bacteroidia and Alphaproteobacteria, increase in abundance. Furthermore, both alpha and beta diversity indicate that the MGCs successfully mimicked the established microbial communities for Riftia Field and Marker 28. It has been suggested that hydrothermal vent Alphaproteobacteria and Gammaproteobacteria are distinct from other deep-sea heterotrophs (92). Transcriptomic studies at the ASHES vent field along the Juan de Fuca Ridge suggested that Gammaproteobacteria may also drive diffuse vent productivity since they are less sulfide-tolerant than Campylobacteria (93, 94). Metagenomic and metaproteomic analysis of inactive hydrothermal sulfides identified Gammaproteobacteria in hydrothermal vent environments as primary producers using RubisCO (95), and subsequent work detected autotrophic Gammaproteobacteria on the same types of samples, where rates of primary productivity were found to be similar to those in diffuse fluids (51). The MGCs had common Gammaproteobacterial genera to hydrothermal vent systems, such as *Marinobacter* and *Halomonas* (96), the autotrophic hydrocarbon oxidizers *Methyloprofundus* and the hydrocarbon degrader *Alcanovorax*, which has been found to be abundant in hydrothermal plumes (97). The MGCs revealed the presence of putative autotrophic Methylomonadaceae, including *Methyloprofundus*, and also *Thiomicrorhabdus*, indicating they are potentially contributors to primary production at the lower temperature sites (98, 99). Additionally, Gammaproteobacteria were found to be important in mature microbial communities, and at a shallow water hydrothermal vent, Gammaproteobacteria were more abundant in the established microbial mats (100).

The MGCs at EPR have an abundance of multiple chemosynthetic taxa that utilize different carbon fixation pathways. Recent work at EPR 9°50′N was able to implicate both CBB and rTCA in measured primary productivity on inactive sulfides by looking at δ^13^C of both lipids and total organic carbon (51). The autotrophic Zetaproteobacteria and Gammaproteobacteria have been shown to fix carbon via the Calvin-Benson-Bassham cycle (21, 62, 101). However, Campylobacteria employ the rTCA pathway for carbon fixation (102). Primary production via rTCA has a distinctly different carbon isotopic signature from carbon fixation via RubisCO (103–105). This mixed autotrophic community should be considered when examining carbon isotopic signatures of lipids of vent organisms or the geological record (106).

Although ASVs were shared between locations, no common ASV was identified as a network hub across the networks of MGCs incubated at different temperatures and for different durations, suggesting community assembly at vents is likely influenced by stochastic processes. Microbial community assembly patterns are poorly understood, but generally can occur in deterministic or stochastic processes. Inter-species interactions and relationships, and environmental parameters guide deterministic processes, whereas stochastic processes depend on random changes, such as colonization, death, dispersal limitation, or speciation (107). Using an experimental system to control biofilm thickness, deterministic processes were found to drive community composition (108). Another controlled laboratory study with a bioreactor showed stochastic assembly as the dominant factor governing microbial community assembly, resulting in functional variations (109). However, the rare OTUs were identified as stochastically assembled in an activated sludge system, whereas the abundant OTUs were more deterministic (110). Stochastic processes may govern community assembly since they are dispersed from hydrothermal vents through water currents or subsurface aquifers (111). In subsurface microbial systems, both stochastic and deterministic processes have been shown to jointly shape community structure, with deterministic environmental filtering becoming more influential under strong geochemical gradients (112). Consistent with this, the geochemistry of vents is a well-supported driver of community structure (38, 52, 113, 114). Further experiments with standardized incubation times would be necessary to determine whether microbial communities at vents are colonized predominantly through a deterministic or stochastic process.

Hydrothermal vent microbial communities at the EPR 9°50′N exhibit dynamic colonization patterns driven by environmental factors, such as temperature, sulfide, and iron availability, with early colonizers shaping subsequent community development. These findings underscore the role of stochastic processes in microbial community assembly and highlight the importance of long-term incubations in better understanding the ecological and biogeochemical functions of vent-associated microorganisms. Further studies should be expanded upon to assess community function and structure with longer-term incubations and parallel collections of established microbial mats.

## Data Availability

All sequence data are available through the NCBI Sequence Read Archive study number SUB1511609 (BioProject: PRJNA1261830).

## SUPPLEMENTAL MATERIAL

Supplemental figures spectrum.00067-26-s0001.pdf Fig. S1 to S9.

Table S1 spectrum.00067-26-s0002.xlsx List of MGCs with their location, duration, and temperature classification.

Table S2 spectrum.00067-26-s0003.xlsx *P*-values for the two-sample *t*-test comparing MGCs incubated at high and low temperature locations.

Table S3 spectrum.00067-26-s0004.xlsx *P*-values for timeframe.

Table S4 spectrum.00067-26-s0005.xlsx Class identification of shared ASVs.

Table S5 spectrum.00067-26-s0006.xlsx Summary of PERMANOVA analyses based on Euclidean distance of ASVs for MGC bacterial communities.

Table S6 spectrum.00067-26-s0007.xlsx Summary of network characteristics.

Table S7 spectrum.00067-26-s0008.xlsx Taxonomy of network hubs for top 1%.

ASM does not own the copyrights to Supplemental Material that may be linked to, or accessed through, an article. The authors have granted ASM a non-exclusive, world-wide license to publish the Supplemental Material files. Please contact the corresponding author directly for reuse.
