# Supplementary material for: Establishing community structure and diversity within hydrothermal vent bacterial communities of the East Pacific Rise at 9°50′N
Source: Microbiol Spectr. 2026 Mar 9;14(4):e00067-26. doi: 10.1128/spectrum.00067-26 (PMC13055247; doi:10.1128/spectrum.00067-26)
Supplement: Supplemental figures — Fig. S1 to S9. [file spectrum.00067-26-s0001.pdf]

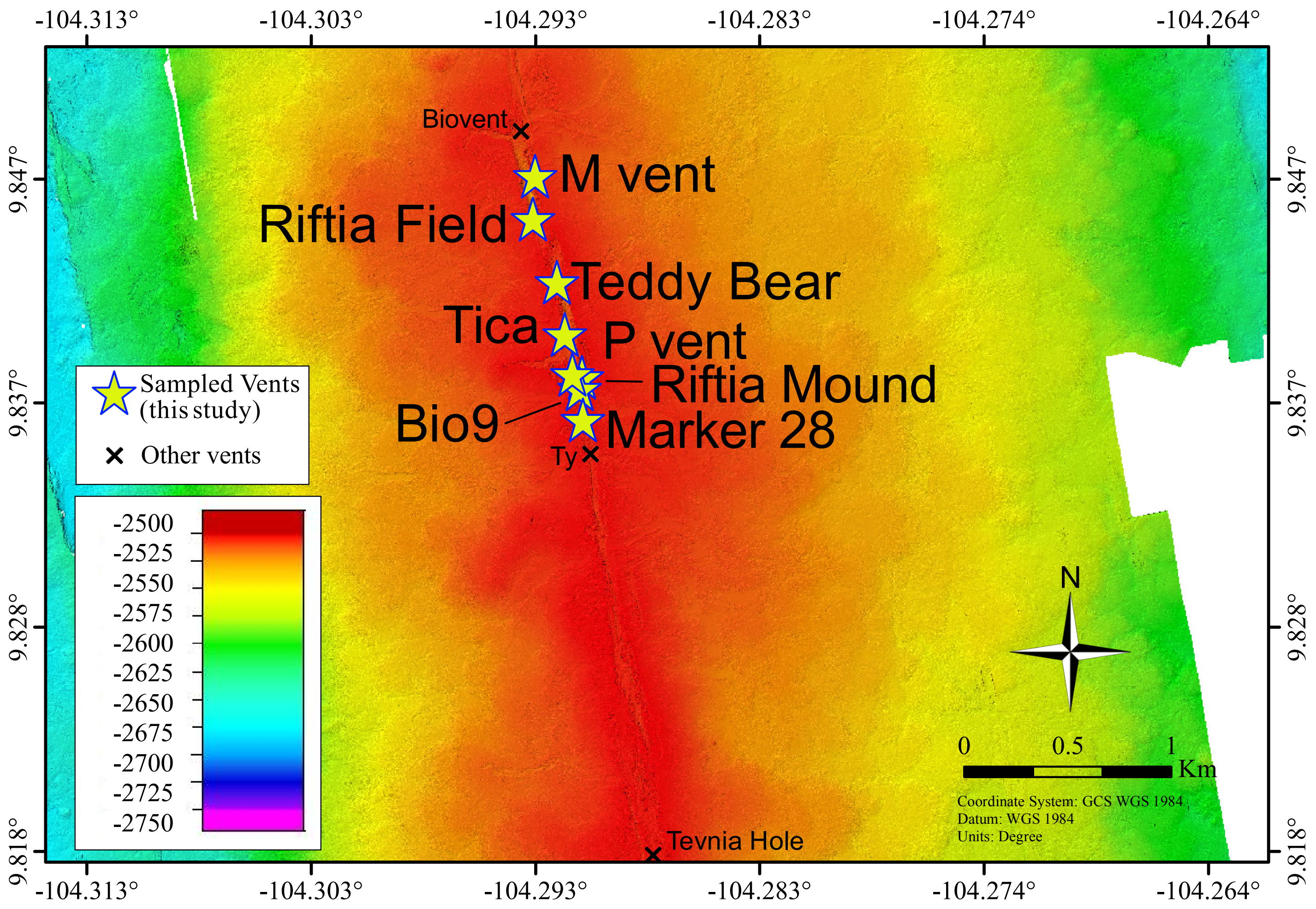

Supplemental Figure 1: Bathymetric map of the sampled region of the East Pacific Rise. Bathymetric data from Wu et al., (2022) and map adapted from Achberger et al. (2024).

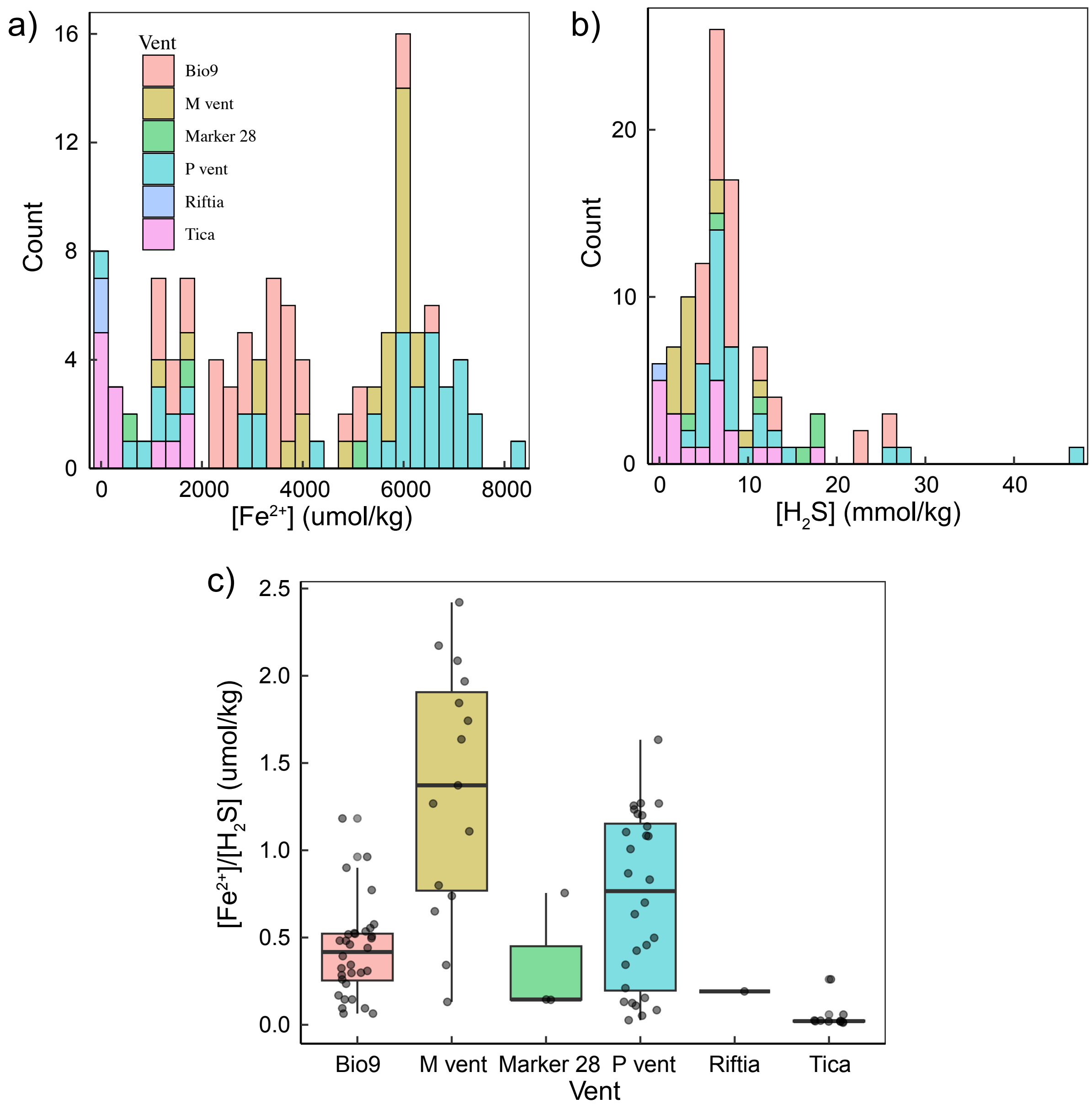

Supplemental Figure 2: The density distribution of dissolved Fe^{2+} (a), H_2S (b), and the molar ratio of dissolved Fe^{2+}/H_2S (c) of fluids sampled from low and high temperature vents, <300 and >300°C, respectively, from the East Pacific Rise 9°N hydrothermal system. Generally, the high temperature fluids are predominantly enriched in dissolved Fe^{2+} relative to H_2S compared to lower temperature fluids. The data are derived from the Diehl and Bach (2020) database.

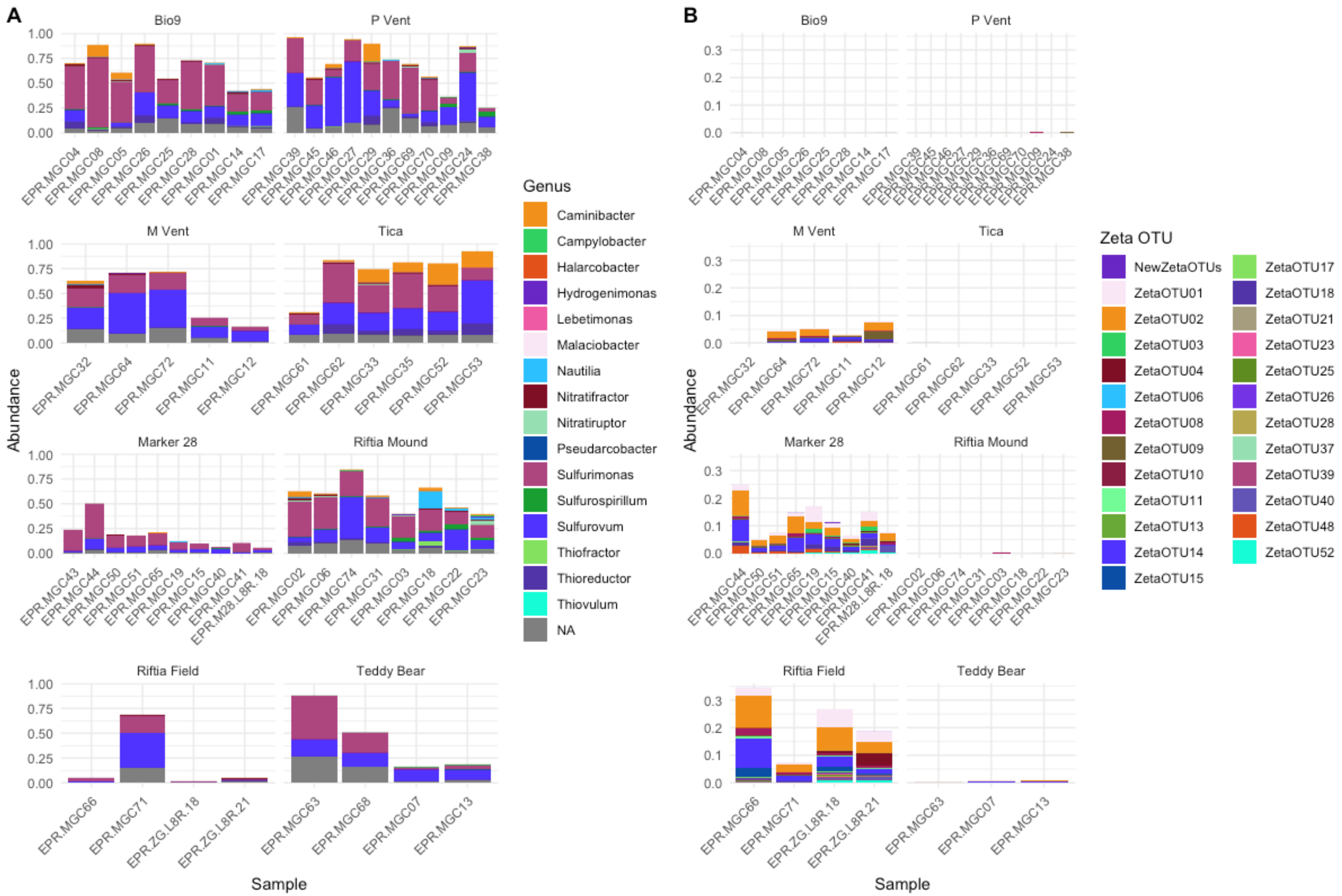

Supplemental Figure 3: Relative abundance of Campylobacteria Genera (A) and Zetaproteobacteria OTUs (B).

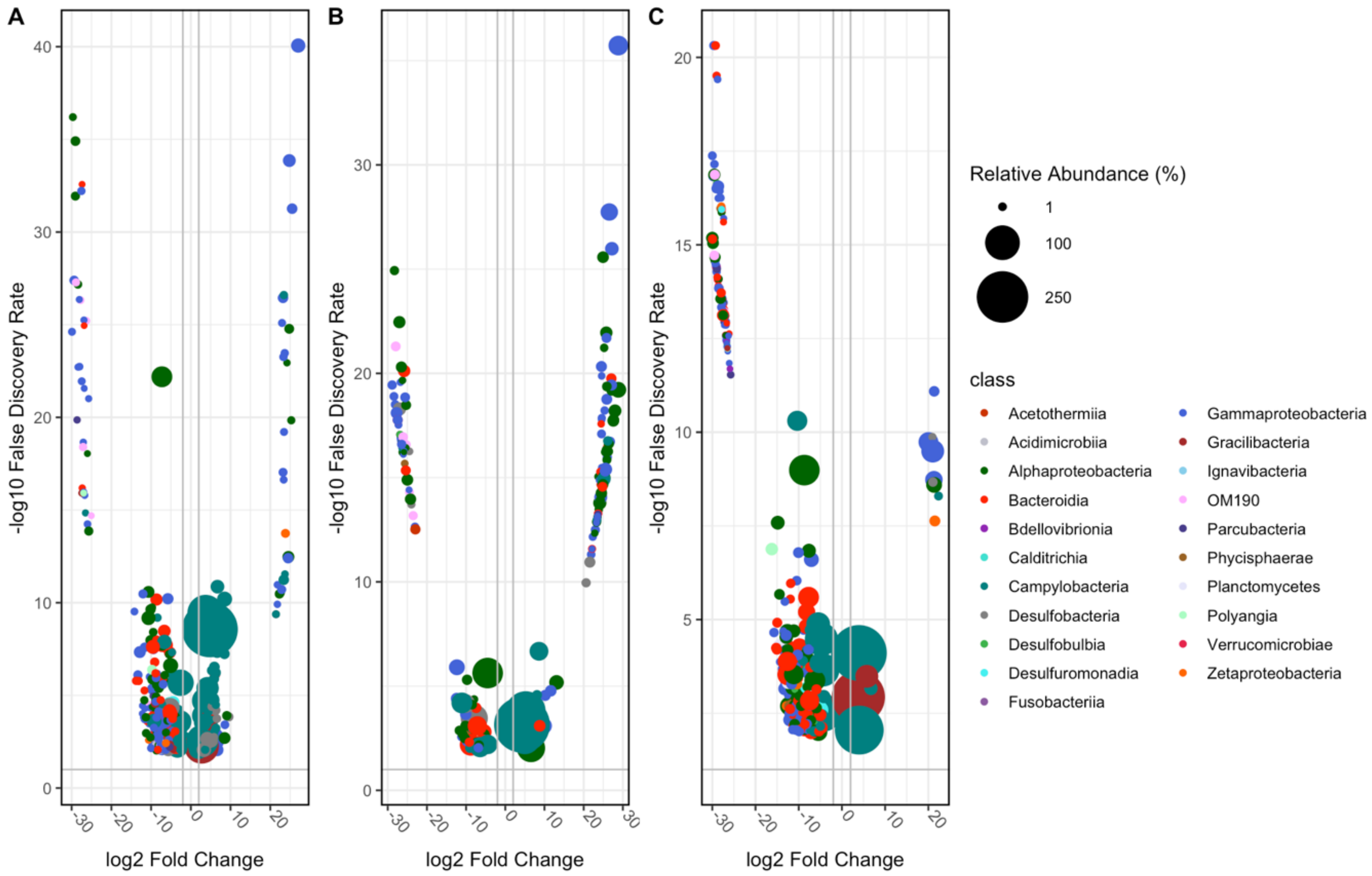

Supplemental Figure 4: DESeq comparisons based on incubation duration, where the long duration is to the left of zero, A) all MGCs, B) Low temperature MGCs, and C) High temperature MGCs. Points are sized by relative abundances across all MGCs in that treatment, and color represents the Class of each ASV.

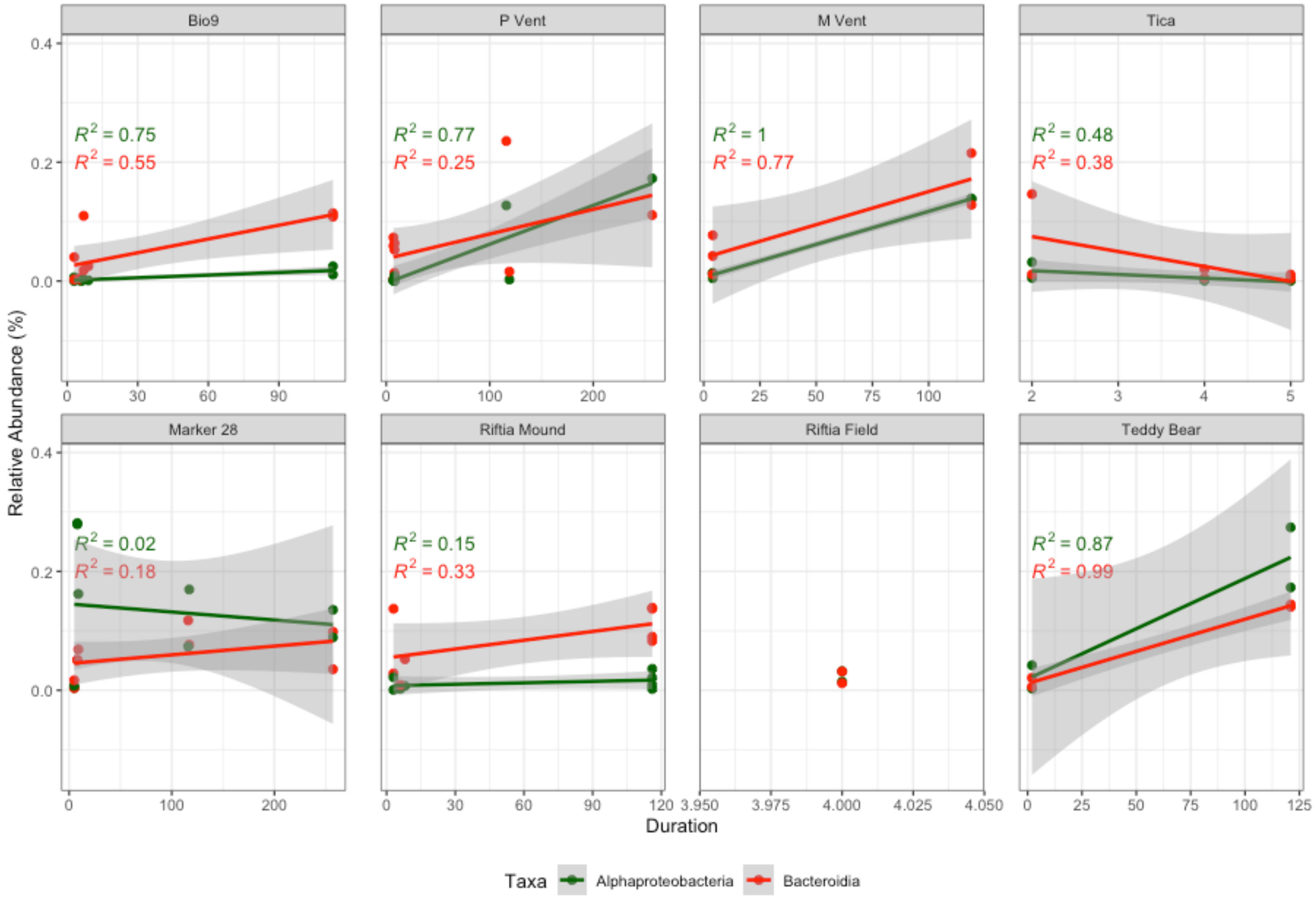

Supplemental Figure 5: Variation in Alphaproteobacteria and Bacteroidia over incubation time at each vent location.

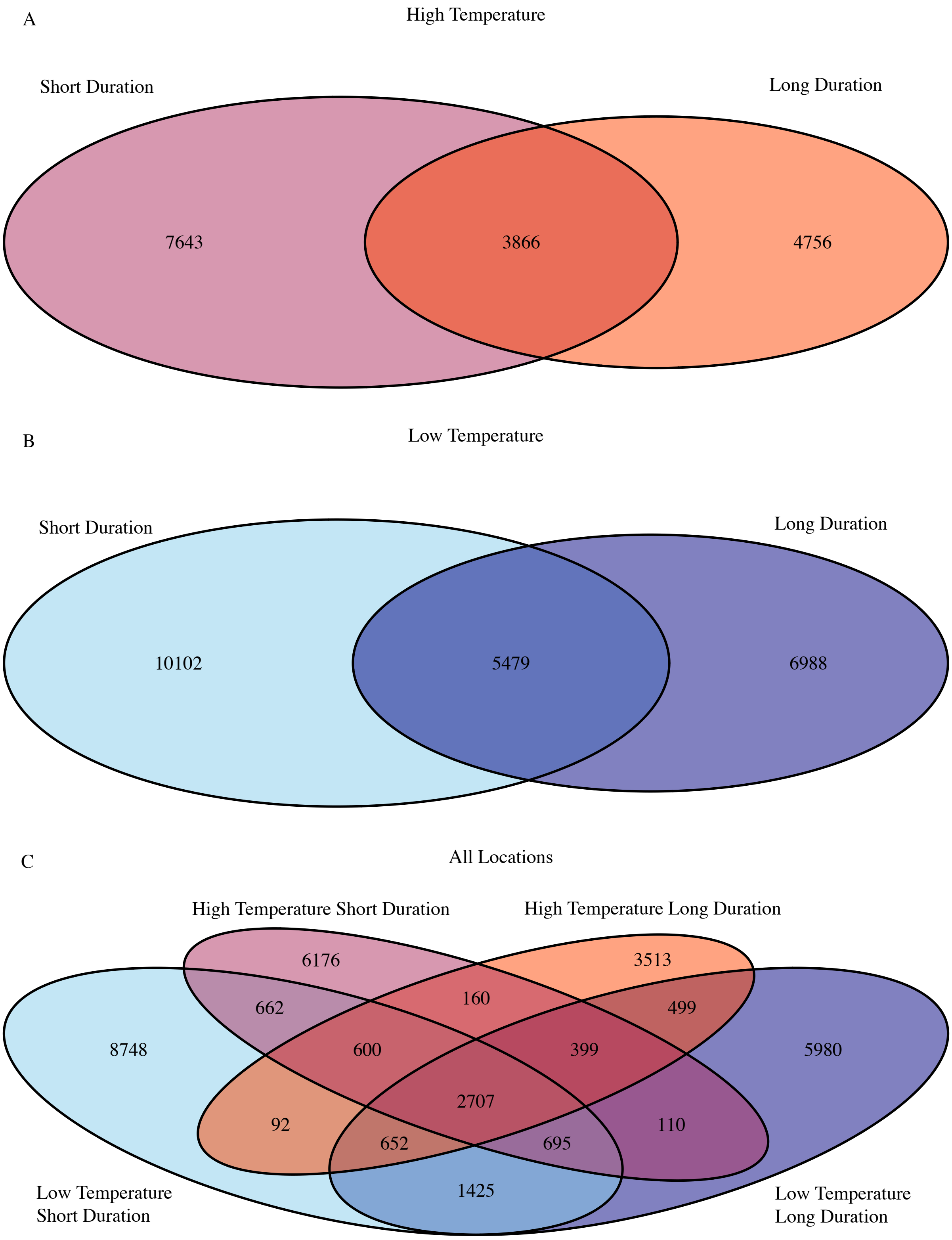

Supplemental Figure 6: Venn diagram of shared ASVs at A) high-temperature locations for short and long durations, B) low-temperature locations for short and long durations, and C) both duration and temperatures.

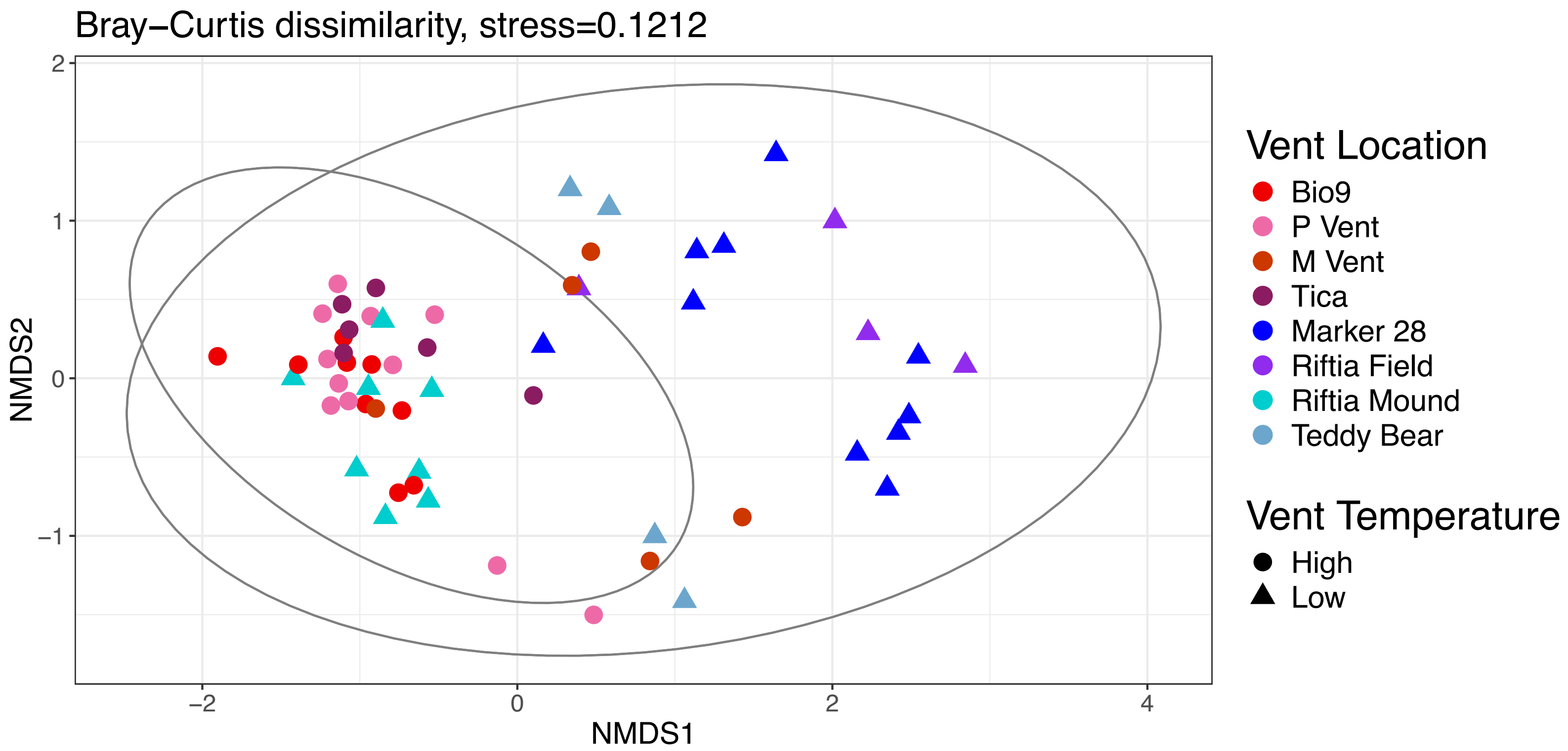

Supplemental Figure 7: Non-metric multidimensional scaling plot generated using Bray-Curtis dissimilarities for the MGC microbial communities. Ellipses represent 95% confidence intervals for the temperature of the vent site.

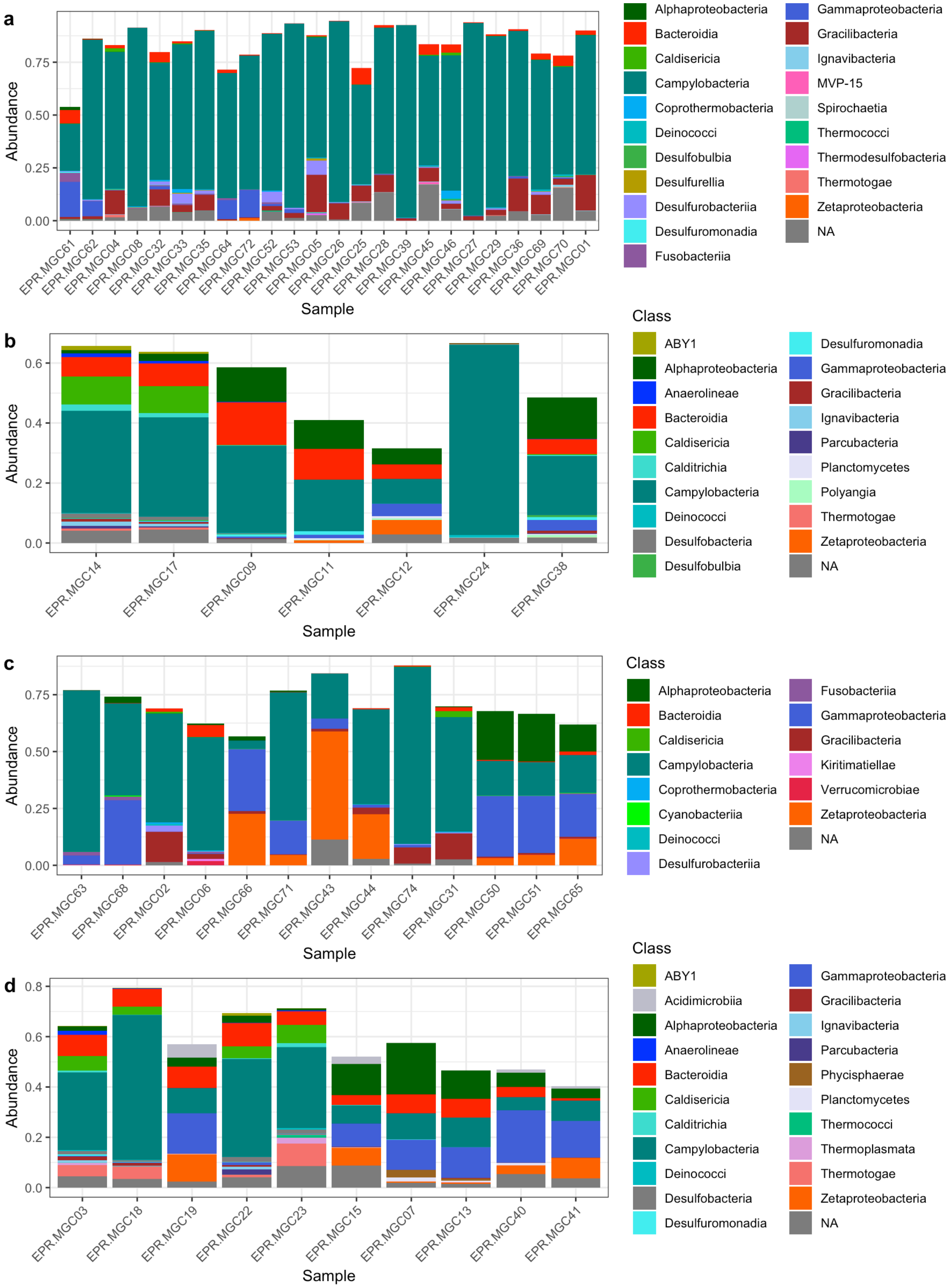

Supplemental Figure 8: Relative abundance of ASVs in network analysis for a) high temperature, short duration b) high temperature, long duration, c) low temperature, short duration and d) low temperature, long duration.

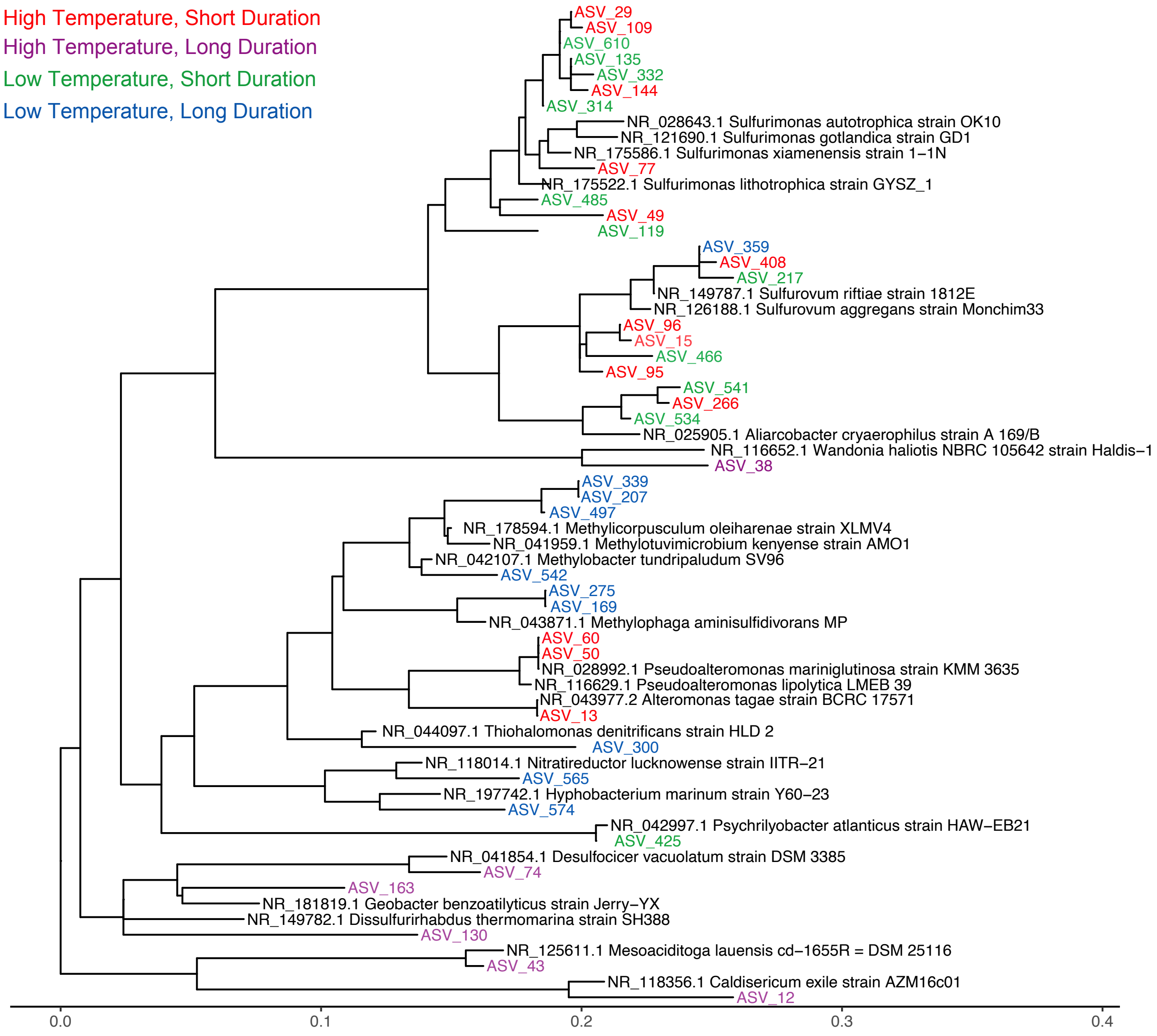

Supplemental Figure 9: Phylogenetic tree of cluster hubs and closest hit to the BLAST Refseq NR database. Branch lengths represent substitutions per site, and the scale bar indicates the expected number of substitutions per site. Tip labels are shown for all taxa.
